# A modified two-layer iteration via a boundary point approach to generalized multivalued pseudomonotone mixed variational inequalities

**DOI:** 10.1186/s13660-017-1482-0

**Published:** 2017-09-12

**Authors:** Ali Mohamed Saddeek

**Affiliations:** Faculty of Science, Department of Mathematics, Assiut University, Assiut, Egypt

**Keywords:** 49J40, 90C33, modified two-layer iteration, multivalued pseudomonotone mapping, generalized mixed variational inequalities, strong convergence, uniformly convex spaces

## Abstract

Most mathematical models arising in stationary filtration processes as well as in the theory of soft shells can be described by single-valued or generalized multivalued pseudomonotone mixed variational inequalities with proper convex nondifferentiable functionals. Therefore, for finding the minimum norm solution of such inequalities, the current paper attempts to introduce a modified two-layer iteration via a boundary point approach and to prove its strong convergence. The results here improve and extend the corresponding recent results announced by Badriev, Zadvornov and Saddeek (Differ. Equ. 37:934-942, 2001).

## Introduction

Let *V* be a real Banach space, $V^{\ast}$ be its dual space, $\Vert \cdot \Vert _{V^{\ast}}$ be the dual norm of the given norm $\Vert \cdot \Vert _{V}$, and $\langle\cdot,\cdot \rangle$ be the duality pairing between $V^{\ast}$ and *V*. Let *M* be a nonempty closed convex subset of *V*. Let $C(V^{\ast})$ be the family of nonempty compact subsets of $V^{\ast}$. Let *H* be a real Hilbert space with the inner product $(\cdot,\cdot )$ and the norm $\Vert \cdot \Vert _{H}$, respectively.

We denote by → and ⇀ strong and weak convergence, respectively. Let $A_{0}: V\rightarrow V^{\ast}$ be a nonlinear single-valued mapping.

### Definition 1.1

see [2–6]

For all $u, \eta\in V$, the mapping $A_{0}:V\rightarrow V^{\ast}$ is said to be as follows:

(i) pseudomonotone, if it is bounded and for every sequence $\{u_{n}\}\subset V$ such that
  $$\begin{aligned} u_{n}\rightharpoonup u \in V\quad\mbox{and}\quad \limsup _{n\rightarrow \infty} \langle A_{0}u_{n},u_{n}-u \rangle\leq0 \end{aligned}$$
  imply
  $$\begin{aligned} \liminf_{n\rightarrow\infty} \langle A_{0}u_{n},u_{n}- \eta \rangle\geq \langle A_{0}\eta,u-\eta\rangle; \end{aligned}$$
(ii) coercive, if there exists a function $\rho: \mathbb {R^{+}}\rightarrow\mathbb{R^{+}}$ with $\lim_{\xi\rightarrow\infty} {\rho(\xi)=+\infty}$ such that
  $$\begin{aligned} \langle A_{0}u,u\rangle\geq \rho\bigl( \Vert u \Vert _{V}\bigr) \Vert u \Vert _{V}; \end{aligned}$$
(iii) potential, if
  $$\begin{aligned} \int^{1}_{0}\bigl(\bigl\langle A_{0} \bigl(t(u+ \eta)\bigr),u+\eta\bigr\rangle -\bigl\langle A_{0}(tu),u\bigr\rangle \bigr) \,dt= \int^{1}_{0}\bigl\langle A_{0}(u+t\eta), \eta\bigr\rangle \,dt; \end{aligned}$$
(iv) bounded Lipschitz continuous, if
  $$\begin{aligned} \Vert A_{0}u-A_{0}\eta \Vert _{V^{\ast}} \leq\mu(R) \Phi\bigl( \Vert u-\eta \Vert _{V}\bigr), \end{aligned}$$
  where $R= \max\{ \Vert u \Vert _{V}, \Vert \eta \Vert _{V}\}$, *μ* is a nondecreasing function on $[0,+\infty)$, and Φ is the gauge function (i.e., it is a strictly increasing continuous function on $[0,+\infty)$ such that $\Phi(0)=0$ and $\lim_{\xi\rightarrow\infty} {\Phi(\xi)=+\infty}$);
(v) uniformly monotone, if there exists a gauge Φ such that
  $$\begin{aligned} \langle A_{0}u-A_{0}\eta,u-\eta\rangle\geq \Phi \bigl( \Vert u-\eta \Vert _{V}\bigr) \Vert u-\eta \Vert _{V}; \end{aligned}$$
(vi) inverse strongly monotone, if there exists a constant $\gamma >0$ such that
  $$\begin{aligned} \langle A_{0}u-A_{0}\eta,u-\eta\rangle\geq \gamma \Vert A_{0}u-A_{0}\eta \Vert _{V}^{2}. \end{aligned}$$

If $\Phi(\xi)=\xi$ and $\mu(R)=\gamma>0$, in (iv), the mapping $A_{0}$ is called *γ*-Lipschitzian mapping, and if there exists $\alpha>0$ such that $\Phi(\xi)=\alpha\xi$, in (v), the mapping $A_{0}$ is called strongly monotone mapping. It is obvious that any inverse strongly monotone mapping is $\frac{1}{\gamma}$-Lipschitzian mapping.

The single-valued pseudomonotone mixed variational inequality problem is formulated as finding a point $u\in M$ such that

1.1 $$\begin{aligned} \langle A_{0}u,\eta-u\rangle+F_{1}(\eta)-F_{1}(u) \geq \langle f,\eta-u\rangle\quad \forall\eta\in M, \end{aligned}$$

where $A_{0}:V\rightarrow V^{\ast}$ is a single-valued pseudomonotone mapping, $F_{1}:V\rightarrow\mathbb{R}\cup \{+\infty\}$ is a proper convex and lower semicontinuous (but, in general, nondifferentiable) functional, and $f\in V^{\ast}$ is a given element.

Problem (1.1) is equivalent to finding $u\in V$ such that

1.2 $$\begin{aligned} 0\in A_{0}u-f+\partial F_{1}(u), \end{aligned}$$

where $\partial F_{1}(u)$ is the subdifferential of $F_{1}$, i.e.,

$$\begin{aligned} \partial F_{1}(u)=\bigl\{ u^{\ast}\in V^{\ast}: F_{1}(\eta)-F_{1}(u)\geq \bigl\langle u^{\ast},\eta-u \bigr\rangle \ \forall\eta\in V\bigr\} . \end{aligned}$$

The interior of the domain of $F_{1}$ is denoted by $\operatorname{int} (D(F_{1}))$.

Such problems appear in many fields of physics (e.g., in hydrodynamics, elasticity or plasticity), more specifically, when describing or analyzing the steady state filtration (see, for example, [1, 7–9] and the references cited therein) and the problem of finding the equilibrium of soft shells (see, for example, [1, 7, 10–12] and the references cited therein).

The existence of at least one solution to problem (1.1) can be guaranteed by imposing pseudomonotonicity and coercivity conditions on the mapping $A_{0}$ (see, for example, [2, 3]).

If $f=0$ and $F_{1}(u)=I_{M}(u)$ $\forall u\in M$, where $I_{M}$ is the indicator functional of *M* defined by $u\in M$ such that $I_{M}(u)= \bigl\{ \scriptsize{ \begin{array}{l@{\quad}l} 0, & u\in M,\\ +\infty,& \mbox{o.w}, \end{array}}$ then problem (1.1) is equivalent to finding $u\in M$ such that

1.3 $$\begin{aligned} \langle A_{0}u,\eta-u\rangle\geq 0\quad \forall\eta\in M, \end{aligned}$$

which is known as the classical variational inequality problem firstly introduced and studied by Stampacchia [13]. Problem (1.3) is equivalent to the following nonlinear operator equation: find $u\in M$ such that

1.4 $$\begin{aligned} A_{0}u=f. \end{aligned}$$

A mapping $J:V\rightarrow V^{\ast}$ is called a duality mapping with gauge function Φ if, for every $u\in V$, $\langle Ju,u \rangle=\Phi( \Vert u \Vert _{V}) \Vert u \Vert _{V}$ and $\Vert Ju \Vert _{V^{\ast }}=\Phi( \Vert u \Vert _{V})$. If $V=H$, then the duality mapping with the gauge function $\Phi(\xi)=\xi$ can be identified with the identity mapping of *H* into itself.

It is well known (see, for example, [3, 14]) that $J(0)=0$, *J* is odd, single-valued, bijective and is uniformly continuous on bounded sets if *V* is a reflexive Banach space and $V^{\ast}$ is uniformly convex; moreover, $J^{-1}$ is also single-valued, bijective, and $JJ^{-1}=I_{V^{\ast}}$, $J^{-1}J=I_{V}$.

Therefore, we always assume that the dual space of a reflexive Banach space is uniformly convex.

### Remark 1.1

see, for example, [15]

The single-valued duality mapping *J* is bounded Lipschitz continuous and uniformly monotone.

In order to find a solution of problem (1.1), Badriev et al. [1] suggested the following two-layer iteration method: for an arbitrary $u_{0}\in M$, define $u_{n+1} \in M$ as follows:

1.5 $$\begin{aligned} \bigl\langle J(u_{n+1}-u_{n}),\eta-u_{n+1}\bigr\rangle +\tau \bigl(F_{1}(\eta)-F_{1}(u_{n+1})\bigr) \geq\tau\langle f-A_{0}u_{n},\eta-u_{n+1}\rangle\quad \forall \eta\in M, \end{aligned}$$

where $\tau>0$ is an iteration parameter and $n\geq0$.

In this way the original variational inequality problem (1.1) is thus reduced to another variational inequality problem involving the duality mapping *J* instead of the original pseudomonotone mapping $A_{0}$. Such a problem can then be solved by known methods (see, for example, [16, 17]).

If $V=H$, then the iteration generated by (1.5) can be written in the following form:

1.6 $$\begin{aligned} (u_{n+1}-u_{n},\eta-u_{n+1})+\tau \bigl(F_{1}(\eta)-F_{1}(u_{n+1})\bigr)\geq\tau (f-A_{0}u_{n},\eta-u_{n+1})\quad \forall \eta\in M, \end{aligned}$$

for an arbitrary $u_{0}\in M$ and $\tau>0$.

In [18], Saddeek and Ahmed considered the following two-layer iteration method for solving the nonlinear operator equation (1.4) in a Banach space *V*:

1.7 $$\begin{aligned} J(u_{n+1}-u_{n})= \tau(f-A_{0}u_{n}),\quad n\geq0, \end{aligned}$$

where $u_{0}$ is an arbitrary point in *M* and $\tau>0$.

In the case when $V=H$, iteration (1.7) can be written as follows:

1.8 $$\begin{aligned} u_{n+1}=u_{n}- \tau(A_{0}u_{n}-f),\quad n \geq0, \end{aligned}$$

for $\tau>0$ and $u_{0}$ is an arbitrary point in *M*.

Saddeek and Ahmed [18] proved some weak convergence theorems of iterations (1.7) and (1.8) for approximating the solution of nonlinear equation (1.4).

Attempts to modify the two-layer iterations (1.7) and (1.8) so that strong convergence is guaranteed have recently been made.

In [19], Saddeek introduced the following modification of (1.8) in a Hilbert space *H* (boundary point method):

1.9 $$\begin{aligned} u_{n+1}=u_{n}-\tau h(u_{n}) (A_{0}u_{n}-f),\quad n\geq0, \end{aligned}$$

where $\tau>0$, $u_{0}$ is an arbitrary point in *M*, and $h:M \rightarrow[0,1]$ is a function defined by He and Zhu [20] as follows:

1.10 $$\begin{aligned} h(u)=\inf\bigl\{ \alpha\in[0,1]: \alpha u\in M\bigr\} \quad \forall u\in M. \end{aligned}$$

He obtained strong convergence results for finding the minimum norm solution of nonlinear equation (1.4).

In [20], He and Zhu have observed that, if $0 \notin M$, calculating $h(u_{n})$ implies determining $h(u_{n})u_{n}$, a boundary point of *M*, so iteration (1.9) is known as the boundary point method.

In [21], Saddeek extended the results of Saddeek [19] to a uniformly convex Banach space and introduced the following modification of the two-layer iteration (1.7) (boundary point method):

1.11 $$\begin{aligned} Ju_{n+1}=Ju_{n}-\tau h(u_{n}) (A_{0}u_{n}-f),\quad n\geq0, \end{aligned}$$

where $\tau>0$, $u_{0}$ is an arbitrary point in *M*, $\tau>0$, and *h* is defined by (1.10).

In [22], Noor introduced and studied the following generalized multivalued pseudomonotone mixed variational inequality problem: find $u\in M$, $w\in A_{0}(u)$ such that

1.12 $$\begin{aligned} \langle w,\eta-u\rangle+F_{1}(\eta)-F_{1}(u) \geq \langle f,\eta-u\rangle \quad\forall\eta\in M, \end{aligned}$$

where $A_{0}:V\rightarrow C(V^{\ast})$ is a multivalued pseudomonotone mapping (see definition below), $F_{1}:V\rightarrow \mathbb{R}\cup\{+\infty\}$ is a functional as above, and $f\in V^{\ast}$ is a given element.

Clearly, problems (1.1) and (1.3) are special cases of problem (1.12).

The set of all $u\in M$ satisfying (1.12) is denoted by $\mathit{SOL}(M,F_{1},A_{0}-f)$.

In [1], Badriev et al. obtained the following weak convergence theorems using the two-layer iteration (1.5).

### Theorem 1.1

see [1], Theorem 1

*Let* *V* *be a real reflexive Banach space with a uniformly convex dual space* $V^{\ast}$, *and let* $J:V \rightarrow V^{\ast}$ *be the duality mapping*. *Let* *M* *be a nonempty closed convex subset of* *V*. *Let* $A_{0}:V \rightarrow V^{\ast}$ *be a pseudomonotone*, *coercive*, *potential*, *and bounded Lipschitz continuous mapping*. *Let* $F_{1}:V\rightarrow\mathbb{R}\cup \{+\infty\}$ *be a proper convex and* *γ*-*Lipschitzian* (*i*.*e*., $\mid F_{1}(u)-F_{1}(\eta)\mid \leq\gamma \Vert u-\eta \Vert _{V}$ $\forall u,\eta\in V, \gamma>0$) *functional*. *Define a functional* $F:V\rightarrow\mathbb{R}\cup\{+\infty\}$ *by*

1.13 $$\begin{aligned} F(u)=F_{0}(u)+F_{1}(u)-\langle f,u\rangle,\qquad F_{0}(u)= \int^{1}_{0}\bigl\langle A_{0}\bigl(t(u)\bigr),u \bigr\rangle \,dt,\quad f\in V^{\ast}. \end{aligned}$$

*Assume also that*

1.14 $$\begin{aligned} 0< \tau< \min \biggl\{ 1,\frac{1}{{\mu}_{0}} \biggr\} ,\quad {\mu}_{0}= \mu \bigl({R}_{0}+\Phi^{-1}({R}_{1}+\gamma)\bigr), \end{aligned}$$

*where*

$$\begin{aligned} {R}_{0}=\sup_{u\in{S}_{0}} \Vert u \Vert _{V},\qquad {R}_{1}=\sup_{u\in {S}_{0}} \Vert A_{0}u-f \Vert _{V^{\ast}},\qquad {S}_{0}=\bigl\{ u\in M:{F}(u)\leq {F}(u_{0})\bigr\} . \end{aligned}$$

*Then the sequence* $\{u_{n}\}$ *defined by* (1.5) *is bounded in* *V*, *and all of its weak limit points are solutions of problem* (1.1).

Badriev et al. [1] have remarked that, due to the reflexivity of *V*, the mixed variational inequality (1.1) is solvable by Theorem 1.1.

In Theorem 1.1, the assumption that *V* is reflexive can be dropped. Indeed, if $V^{\ast}$ is uniformly convex, then *V* is uniformly smooth (and hence *V* is reflexive).

### Theorem 1.2

see [1], Theorem 2

*Let* $V=H$ *be a real Hilbert space*, *and let* *M* *be a nonempty closed convex subset of* *H*. *Let* $A_{0}:H \rightarrow H$ *be a pseudomonotone*, *coercive*, *potential*, *and inverse strongly monotone mapping*. *Let* $F_{i}:H\rightarrow\mathbb{R}\cup\{+\infty\}$, $i=0,1$, *be the same as in Theorem* 1.1.

*Then the sequence* $\{u_{n}\}$ *defined by* (1.6) *with* $0<\tau<\tau_{0}=2\gamma$ *converges weakly in* *H* *to a solution of problem* (1.1).

Some attempts to prove the weak convergence of the whole sequence in the framework of Banach spaces have been made by Saddeek and Ahmed [23] and Saddeek [24, 25].

Although the above mentioned theorems and all their extensions are unquestionably interesting, only weak convergence theorems are obtained unless very strong assumptions are made.

This suggests an important question: can the two-layer iteration method (1.5) be modified to prove its strong convergence to the minimum norm solution of problem (1.12).

In this paper, inspired by [20, 21], and [22], a generalized multivalued pseudomonotone mixed variational inequality is considered, and a modified two-layer iteration via a boundary point approach to find the minimum norm solution of such inequalities is introduced, and its strong convergence is proved in the framework of uniformly convex spaces. The results obtained in this paper improve and generalize the corresponding recent results announced by [1].

## Definitions and preliminary

### Definition 2.1

see [5, 26, 27]

A multivalued mapping $A_{0}:V\rightarrow C(V^{\ast})$ is called

(i) pseudomonotone, if it is bounded and, for every sequence $\{u_{n}\}\subset V$, $\{w_{n}\}\subset A_{0}(u_{n})$, the conditions
  $$\begin{aligned} u_{n}\rightharpoonup u \in V\quad\mbox{and}\quad \limsup _{n\rightarrow \infty} \langle w_{n},u_{n}-u\rangle\leq0 \end{aligned}$$
  imply that for every $\eta\in V$ there exists $w \in A_{0}(u)$ such that
  $$\begin{aligned} \liminf_{n\rightarrow\infty} \langle w_{n},u_{n}- \eta \rangle\geq \langle w,u-\eta\rangle; \end{aligned}$$
(ii) coercive, if there exists a function $\rho: \mathbb {R^{+}}\rightarrow\mathbb{R^{+}}$ with $\lim_{\xi\rightarrow\infty} {\rho(\xi)=+\infty}$ such that
  $$\begin{aligned} \langle w,u\rangle\geq \rho\bigl( \Vert u \Vert _{V} \bigr) \Vert u \Vert _{V} \quad\forall u\in V, w\in A_{0}(u); \end{aligned}$$
(iii) potential, if
  $$\begin{aligned} \int^{1}_{0}\bigl(\bigl\langle w^{1},u+ \eta\bigr\rangle -\bigl\langle w^{2},u\bigr\rangle \bigr) \,dt= \int^{1}_{0}\bigl\langle w^{3},\eta\bigr\rangle \,dt \end{aligned}$$
  for all $u,\eta\in V$, $w^{1}\in A_{0}(t(u+\eta))$, $w^{2}\in A_{0}(t u)$, $w^{3}\in A_{0}(u+t\eta)$, $t\in[0,1]$;
(iv) bounded Lipschitz continuous, if
  $$\begin{aligned} \Vert w-\acute{w} \Vert _{V^{\ast}} \leq\mu(R) \Phi\bigl( \Vert u-\eta \Vert _{V}\bigr) \end{aligned}$$
  for all $u,\eta\in V$, $w\in A_{0}(u)$, $\acute{w}\in A_{0}(\eta)$, where $\mu(R)$ and $\Phi(\xi)$ as above;
(v) inverse strongly monotone, if there exists a constant $\gamma >0$ such that
  $$\begin{aligned} \langle w-\acute{w},u-\eta\rangle\geq \gamma \Vert w-\acute{w} \Vert _{V}^{2} \end{aligned}$$
  for all $u,\eta\in V$, $w\in A_{0}(u)$, $\acute{w}\in A_{0}(\eta)$.

Definition 2.1 is an extension of Definition 1.1((i)-(iv), (vi)) of single-valued mappings to multivalued mappings.

Let $G_{1}:M\times V^{\ast} \rightarrow\mathbb{R}\cup\{+\infty\}$ be a functional defined as follows:

2.1 $$\begin{aligned} G_{1}(u,J\eta)= \Vert u \Vert _{V}^{2}-2 \langle J\eta,u\rangle+ \Vert J\eta \Vert _{ { V^{\ast}}}^{2}+2F_{1}(u), \end{aligned}$$

where $u\in M$, $\eta\in V$, $J\eta\in V^{\ast}$.

### Definition 2.2

see, for example, [28]

The mapping $\Pi^{F_{1}}_{M}: V\rightarrow C(M)$ is called generalized $F_{1}$-projection mapping if $\Pi^{F_{1}}_{M}(\eta)=\arg\min_{u\in M} G_{1}(u,J\eta )$, $\forall\eta\in V$.

If $V=H$ and $F_{1}(u)=0$ $\forall u\in M$, then (2.1) reduces to the following simple form:

$$\begin{aligned} G_{1}(u,J\eta)= \Vert u-\eta \Vert _{H}^{2},\quad \forall u\in M, \eta\in H, \end{aligned}$$

and the generalized $F_{1}$-projection reduces to the projection $\Pi_{M}$ from *H* to $C(M)$.

The following two lemmas are also useful in the sequel.

### Lemma 2.1

see [28]

*The generalized* $F_{1}$-*projection* $\Pi^{F_{1}}_{M}(\eta)$ *has the following properties*:

(i) $\Pi^{F_{1}}_{M}(\eta)$ *is a nonempty closed convex subset of* *M* *for all* $\eta\in V$;
(ii) *for all* $\eta\in V$, $\bar{u}\in\Pi^{F_{1}}_{M}(\eta)$ *if and only if*
  $$\begin{aligned} \langle J\eta-J\bar{u},\bar{u}-v\rangle+ (F_{1}(v)-F_{1}( \bar{u})\geq0\quad \forall v\in M; \end{aligned}$$
(iii) *if* *V* *is strictly convex*, *then* $\Pi^{F_{1}}_{M}(\eta)$ *is a single*-*valued mapping*.
*Let* $G_{2}:V\times V \rightarrow\mathbb{R^{+}}\cup\{0\}$ *be a functional defined as follows*:
  2.2 $$\begin{aligned} G_{2}(u,\eta)= \Vert u \Vert _{V}^{2}-2 \langle J\eta,u\rangle+ \Vert \eta \Vert _{ V}^{2}, \quad\forall u, \eta\in V. \end{aligned}$$

### Lemma 2.2

see [29]

*Let* *V* *be a real Banach space with a uniformly convex dual space* $V^{\ast}$, *let* *M* *be a nonempty closed convex subset of* *V*, *and let* $\eta\in V$, $\bar{u}\in\Pi^{F_{1}}_{M}(\eta)$. *Then*

(i) $G_{2}(u,\bar{u})+G_{2}(\bar{u},\eta)\leq G_{2}(u,\eta)$ $\forall u\in M$;
(ii) *for* $u, \eta\in V$, $G_{2}(u,\eta)=0$ *iff* $u=\eta$.

*A Banach space* *V* *is said to have the Kadec*-*Klee property* (*see*, *for example*, [30]) *if*, *for every sequence* $\{u_{n}\}$ *in* *V* *with* $u_{n}\rightharpoonup u$ *and* $\Vert u_{n} \Vert _{V}\rightarrow \Vert u \Vert _{V}$ *together imply that* $\lim_{n\rightarrow\infty} \Vert u_{n}-u \Vert _{V}=0$.

*Every Hilbert space is uniformly convex*, *and every uniformly convex Banach space has the Kadec*-*Klee property*.

## Main results

In this section, we propose a modification of the two-layer iteration method (1.5) by the boundary point method to establish strong convergence theorems of the modified iteration for finding the minimum norm solution of the following generalized pseudomonotone mixed variational inequality in uniformly convex spaces: find $u\in M$, $w\in A_{0}(u)$ such that

3.1 $$\begin{aligned} \bigl\langle h (w),\eta-u\bigr\rangle +F_{1}(\eta)-F_{1}(u) \geq \langle f,\eta-u\rangle \quad\forall\eta\in M, \end{aligned}$$

where $A_{0}$, $F_{1}$, *f* are defined as above and *h* is a positive constant.

### The modified two-layer iteration

For an arbitrary point $u_{0} \in M$, define $u_{n+1}\in M$ as follows:

3.2 $$\begin{aligned} \langle Ju_{n+1}-Ju_{n},\eta-u_{n+1}\rangle+\tau \bigl(F_{1}(\eta)-F_{1}(u_{n+1})\bigr)\geq\tau\bigl\langle f-h(u_{n})w_{n},\eta-u_{n+1}\bigr\rangle \quad\forall \eta\in M, \end{aligned}$$

where $\tau>0$ is the iteration parameter, $n\geq0$, *J* is the duality mapping, $w_{n}\in A_{0}(u_{n})$ and *h* is defined by (1.10).

For $M=V$, $F_{1}(u)=0$ $\forall u\in M$, and $\eta=u_{n+1}\pm z$, $z\in M$, (3.2) is equivalent to

3.3 $$\begin{aligned} Ju_{n+1}=Ju_{n}-\tau\bigl(h(u_{n})w_{n}-f \bigr),\quad \forall n\geq0, \end{aligned}$$

where $w_{n}\in A_{0}(u_{n})$, and *τ*, *J*, *h* are defined as above.

Observe that iteration (3.3) is a modification and generalization of iterations (1.11) and (1.9).

If $V=H$, $A_{0}$ is a single-valued mapping in (3.3) and $h(u_{n})=1$ $\forall n\geq0$, we have iteration (1.8).

Iteration (3.3) can be considered as a modified method for solving the following operator inclusion problem: find $u\in V$ such that

3.4 $$\begin{aligned} f\in A_{0} u,\quad f\in V^{\ast}. \end{aligned}$$

For each $u\in V$, $w^{2}\in A_{0}(t u)$, let $\tilde{F}:V\rightarrow\mathbb{R}\cup\{+\infty\}$ be a functional defined by

3.5 $$\begin{aligned} \tilde{F}(u)=\tilde{F}_{0}(u)+F_{1}(u)-\langle f,u \rangle, \qquad \tilde{F}_{0}(u)= \int^{1}_{0}\bigl\langle h(u)w^{2},u\bigr\rangle \,dt,\quad f\in V^{\ast}. \end{aligned}$$

Let us assume also that

3.6 $$\begin{aligned}& \tilde{{R}_{0}}=\sup_{u\in \tilde{{S}}_{0}} \Vert u \Vert _{V}, \qquad \tilde{{R}_{1}}=\sup_{u\in \tilde{{S}_{0}}} \bigl\Vert h(u)w-f \bigr\Vert _{V^{\ast}}, \\& \tilde{{S}_{0}}=\bigl\{ u\in M: \tilde{F}(u)\leq\tilde{F}(u_{0})\bigr\} , \end{aligned}$$

where $w\in A_{0}(u)$.

Let $\tilde{\mu}_{0}$ be a positive constant such that

3.7 $$\begin{aligned} \tilde{{\mu}_{0}}= \mu\bigl(2\tilde{R}_{0}+ \Phi^{-1}(\tilde{{R}_{1}}+\gamma)\bigr). \end{aligned}$$

#### Theorem 3.1

*Let* *V* *be a real uniformly convex Banach space with a uniformly convex dual space* $V^{\ast}$, $J:V \rightarrow V^{\ast}$ *be the duality mapping*, *and let* *M* *be a nonempty closed convex subset of* *V*. *Let* $A_{0}:V \rightarrow C(V^{\ast})$ *be a multivalued mapping*. *Suppose that* $A_{0}$ *is pseudomonotone*, *coercive*, *potential*, *and bounded Lipschitz continuous mapping*. *Let* $F_{1}:V\rightarrow\mathbb{R}\cup\{+\infty\}$ *be a proper convex* (*not necessarily differentiable*) *and* *γ*-*Lipschitzian functional with* $M\subset \operatorname{int} (D(F_{1}))$. *Let* *F̃*, $\tilde{{R}_{0}}$, $\tilde{{R}_{1}}$, $\tilde{{S}}_{0}$, *and* $\tilde{\mu}_{0}$ *be defined by* (3.5), (3.6), *and* (3.7). *Assume that* $0<\tau=\min\{1,\frac{1}{\tilde{\mu}_{0}}\}$. *Let* $\{h(u_{n})\}$ *be an increasing and bounded real sequence in* $[0,1]$.

*Then*, *for an arbitrary* $u_{0}=u\in M$, *the sequence* $\{u_{n}\}$ *defined by* (3.2) *converges strongly to* $\tilde{u}=\Pi_{\mathit{SOL}(M,F_{1},h(w)-f)}^{F_{1}}{0}$ (*i*.*e*., *the minimum norm element in* $\mathit{SOL}(M,F_{1},h(w)-f)$).

#### Proof

Since $F_{1}$ is supposed to be convex and *γ*-Lipschitzian, and $A_{0}$ is coercive and bounded, it results from [1] and [2] that $F_{1}$ is weakly lower semicontinuous and *F̃* is coercive; moreover, $\tilde{R}_{0}<+\infty$ and $\tilde{R}_{1}<+\infty$. Hence $\tilde{\mu}_{0}<+\infty$. This means that the iterative sequence (3.2) is well defined. □

Now we divide the proof into steps.

*Step* 1. We prove that $\{u_{n}\}$ is bounded. To this end, it suffices to prove that

3.8 $$\begin{aligned} \{u_{n}\}\subset\tilde{S}_{0},\qquad \Vert u_{n} \Vert _{V}\leq\tilde {R}_{0},\quad n\geq 0. \end{aligned}$$

Let us prove (3.8) by induction on *n*. For $n=0$, we have $u_{0}\in \tilde{S}_{0}$. Suppose now that $u_{n}\in\tilde{S}_{0}$. We will show that $u_{n+1}\in\tilde{S}_{0}$.

Setting $\eta=u_{n}$ in (3.2) and taking into account that the functional $F_{1}$ is *γ*-Lipschitzian and *J* is uniformly monotone, and the inequality $\tau\leq1$, we obtain

3.9 $$\begin{aligned} \Phi\bigl( \Vert u_{n+1}-u_{n} \Vert _{V}\bigr) \Vert u_{n+1}-u_{n} \Vert _{V} \leq& \langle Ju_{n+1}-Ju_{n},u_{n+1}-u_{n} \rangle \\ \leq& \tau \bigl[\bigl\langle f-h(u_{n})w_{n}, u_{n+1}-u_{n}\bigr\rangle +F_{1}(u_{n})-F_{1}(u_{n+1}) \bigr] \\ \leq& [\tilde{R}_{1}+\gamma ] \Vert u_{n+1}-u_{n} \Vert _{V}. \end{aligned}$$

Now, using (3.9) together with the strict monotonicity of Φ, we have

3.10 $$\begin{aligned} \Vert u_{n+1}-u_{n} \Vert _{V}\leq \Phi^{-1}( \tilde{R}_{1}+\gamma). \end{aligned}$$

Furthermore, it follows from the bounded Lipschitz continuity of $A_{0}$ that, for any $t\in[0,1]$, $w_{n}\in A_{0}u_{n}$, $w_{n}^{3}\in A_{0}(u_{n+1}+t(u_{n}-u_{n+1}))$

3.11 $$\begin{aligned} \bigl\vert \bigl\langle w_{n}^{3}-w_{n},u_{n+1}-u_{n} \bigr\rangle \bigr\vert \leq& \mu(R_{\ast})\Phi\bigl( \bigl\Vert (1-t) (u_{n+1}-u_{n}) \bigr\Vert _{V}\bigr) \Vert u_{n+1}-u_{n} \Vert _{V} \\ \leq& \mu(R_{\ast})\Phi\bigl( \Vert u_{n+1}-u_{n} \Vert _{V}\bigr) \Vert u_{n+1}-u_{n} \Vert _{V}, \end{aligned}$$

where $R_{{\ast}}=\max\{ \Vert u_{n+1}+t(u_{n}-u_{n+1}) \Vert _{V}, \Vert u_{n} \Vert _{V}\}$.

Since

3.12 $$\begin{aligned} \bigl\Vert u_{n+1}+t(u_{n}-u_{n+1}) \bigr\Vert _{V}- \Vert u_{n} \Vert _{V}\leq \bigl\Vert (1-t) (u_{n+1}-u_{n}) \bigr\Vert _{V}\leq \Vert u_{n+1}-u_{n} \Vert _{V}, \end{aligned}$$

it follows from the definition of $R_{\ast}$ that

3.13 $$\begin{aligned} R_{\ast}\leq2\tilde{R}_{0}+\Phi^{-1}( \tilde{R}_{1}+\gamma). \end{aligned}$$

Since *μ̃* is an increasing function, we must have

3.14 $$\begin{aligned} \tilde{\mu}(R_{\ast})\leq\tilde{\mu}_{0}. \end{aligned}$$

Consequently, it follows from (3.11) and (3.14) that

3.15 $$\begin{aligned} - \bigl\vert \bigl\langle w_{n}^{3}-w_{n},u_{n+1}-u_{n} \bigr\rangle \bigr\vert \geq& -\tilde{\mu}_{0}\Phi\bigl( \Vert u_{n+1}-u_{n} \Vert _{V}\bigr) \Vert u_{n+1}-u_{n} \Vert _{V}. \end{aligned}$$

Moreover, since $A_{0}$ is potential, we have

3.16 $$\begin{aligned} \tilde{F}(u_{n})-\tilde{F}(u_{n+1}) =& \int_{0}^{1}\bigl\langle h(u_{n})w_{n}^{3},u_{n}-u_{n+1} \bigr\rangle \,dt-\langle f,u_{n}-u_{n+1}\rangle+{F}_{1}(u_{n})-{F}_{1}(u_{n+1}) \\ =& \int_{0}^{1}\bigl\langle h(u_{n}) \bigl(w_{n}^{3}-w_{n}\bigr),u_{n}-u_{n+1} \bigr\rangle \,dt-\bigl\langle f-h(u_{n})w_{n},u_{n}-u_{n+1} \bigr\rangle \\ &{} +F_{1}(u_{n})-F_{1}(u_{n+1}) \\ \geq&- \int_{0}^{1} \bigl\vert \bigl\langle h(u_{n}) \bigl(w_{n}^{3}-w_{n} \bigr),u_{n}-u_{n+1}\bigr\rangle \bigr\vert \,dt+ { \tau}^{-1} \bigl[\tau\bigl\langle f-h(u_{n})w_{n},u_{n+1}-u_{n} \bigr\rangle \\ &{}+{F}_{1}(u_{n})-{F}_{1}(u_{n+1}) \bigr]. \end{aligned}$$

Setting $\eta=u_{n}$ in (3.2) and using the uniform monotonicity of *J*, (3.15), (3.16), it results that

3.17 $$\begin{aligned} \tilde{F}(u_{n})-\tilde{F}(u_{n+1}) \geq& -\tilde{ \mu}_{0}\Phi\bigl( \Vert u_{n+1}-u_{n} \Vert _{V}\bigr) \Vert u_{n+1}-u_{n} \Vert _{V} \\ &{} +{\tau}^{-1} \bigl\langle J(u_{n+1})-J(u_{n}),u_{n+1}-u_{n} \bigr\rangle \\ \geq& \lambda\Phi\bigl( \Vert u_{n+1}-u_{n} \Vert _{V}\bigr) \Vert u_{n+1}-u_{n} \Vert _{V},\quad\lambda={\tau}^{-1}-\tilde{\mu}_{0}>0. \end{aligned}$$

This implies that $\tilde{F}(u_{n+1})\leq\tilde{F}(u_{n})\leq \tilde{F}(u_{0})$ and so $u_{n+1}\in\tilde{S}_{0}$. So $\{u_{n}\}$ is bounded.

*Step* 2. We prove that $\lim_{n\rightarrow\infty} \Vert u_{n+1}-u_{n} \Vert _{V}=0$ and $\lim_{n\rightarrow\infty} \Vert Ju_{n+1}-Ju_{n} \Vert _{V^{\ast}}=0$.

It follows from (3.17) that the sequence $\{\tilde{F}(u_{n})\}$ is bounded and monotone, and thus we have that $\lim_{n\rightarrow \infty} \tilde{F}(u_{n})$ exists. This together with (3.17) implies that

3.18 $$\begin{aligned} \lim_{n\rightarrow\infty} \lambda \Phi\bigl( \Vert u_{n+1}-u_{n} \Vert _{V}\bigr) \Vert u_{n+1}-u_{n} \Vert _{V}=0. \end{aligned}$$

Since Φ is continuous and strictly increasing, it follows from (3.18) that

3.19 $$\begin{aligned} \lim_{n\rightarrow\infty} \Vert u_{n+1}-u_{n} \Vert _{V}=0. \end{aligned}$$

Since *J* is bounded Lipschitz continuous, Φ is continuous and $\Phi(0)=0$, it follows from (3.19) that

3.20 $$\begin{aligned} \lim_{n\rightarrow\infty} \Vert Ju_{n+1}-Ju_{n} \Vert _{V^{\ast}}=0. \end{aligned}$$

*Step* 3. We show that there exists a subsequence $\{u_{n_{k}}\}$ of $\{u_{n}\}$ such that $u_{n_{k}}\rightharpoonup \bar{u} \in V$, $\lim_{k\rightarrow\infty}{F_{1}(u_{n_{k}})}\geq F_{1}(\bar{u})$, and $\limsup_{k\rightarrow\infty} h(u_{n_{k}})\langle w_{n_{k}},u_{n_{k}}-\bar{u}\rangle\leq0$.

Since $\{u_{n}\}$ is bounded and *V* is reflexive, we can choose a subsequence $\{u_{n_{k}}\}$ of $\{u_{n}\}$ such that $u_{n_{k}}\rightharpoonup\bar{u} \in V$ as $k\rightarrow\infty$.

This together with the weak lower semicontinuity of $F_{1}$ implies that $\lim_{k\rightarrow\infty}{F_{1}(u_{n_{k}})}\geq F_{1}(\bar{u})$.

Since $F_{1}$ is *γ*-Lipschitzian, $\{h(u_{n})\}\subset[0,1]$, it follows from (3.2) that, for arbitrary $\eta\in M$,

3.21 $$\begin{aligned} h(u_{n_{k}})\langle w_{n_{k}},u_{n_{k}}-\eta \rangle =&h(u_{n_{k}})\langle w_{n_{k}},u_{n_{k}}-u_{n_{k+1}} \rangle+h(u_{n_{k}})\langle w_{n_{k}},u_{n_{k+1}}-\eta\rangle \\ \leq& h(u_{n_{k}})\langle w_{n_{k}},u_{n_{k}}-u_{n_{k+1}} \rangle+\tau^{-1}\langle Ju_{n_{k+1}}-Ju_{n_{k}}, \eta-u_{n_{k+1}}\rangle \\ & {}+\bigl(F_{1}(\eta )-F_{1}(u_{n_{k}}) \bigr)+\bigl(F_{1}(u_{n_{k}})-F_{1}(u_{n_{k+}}) \bigr) \\ &{}+\langle f,u_{n_{k+1}}-u_{n_{k}}\rangle+\langle f,u_{n_{k}}-\eta\rangle \\ \leq& \bigl( \Vert w_{n_{k}} \Vert _{V^{\ast}}+ \Vert f \Vert _{V^{\ast}}+\gamma\bigr) \Vert u_{n_{k+1}}-u_{n_{k}} \Vert _{V} +\tau^{-1} \bigl[ \Vert Ju_{n_{k+1}}-Ju_{n_{k}} \Vert _{V^{\ast}} \\ & {}\times \Vert \eta-u_{n_{k+1}} \Vert _{V} \bigr] + \bigl(F_{1}(\eta)-F_{1}(u_{n_{k}})\bigr)+\langle f,u_{n_{k}}-\eta\rangle \\ \leq& C_{\eta}\bigl( \Vert Ju_{n_{k+1}}-Ju_{n_{k}} \Vert _{V^{\ast}}+ \Vert u_{n_{k+1}}-u_{n_{k}} \Vert _{V}\bigr) \\ &{} +\bigl(F_{1}(\eta)-F_{1}(u_{n_{k}})\bigr)+\langle f,u_{n_{k}}-\eta\rangle, \end{aligned}$$

where $C_{\eta}$ is a positive constant depending on *η*.

Setting $\eta=\bar{u}$ in (3.21) and using the weak lower semicontinuity of $F_{1}$, (3.19), (3.20), we have

3.22 $$\begin{aligned} \limsup_{k\rightarrow \infty} h(u_{n_{k}})\langle w_{n_{k}},u_{n_{k}}-\bar{u}\rangle \leq& \limsup _{k\rightarrow \infty}C_{\bar{u}}\bigl( \Vert Ju_{n_{k+1}}-Ju_{n_{k}} \Vert _{V^{\ast}}+ \Vert u_{n_{k+1}}-u_{n_{k}} \Vert _{V}\bigr) \\ &{} + \limsup_{k\rightarrow \infty}\bigl(F_{1}( \bar{u})-F_{1}(u_{n_{k}})\bigr)+ \limsup_{k\rightarrow \infty} \langle f,u_{n_{k}}-\bar{u}\rangle \\ \leq& 0. \end{aligned}$$

*Step* 4. We show that $\bar{u}\in \mathit{SOL}(M,F_{1},h(w)-f)$.

Since $\{h(u_{n})\}\subset[0,1]$ is bounded and monotone increasing, it follows that

3.23 $$\begin{aligned} \lim_{n\rightarrow \infty} h(u_{n})=h >0. \end{aligned}$$

By (3.19)-(3.23), the lower semicontinuity of $F_{1}$ and by the pseudomonotonicity of $A_{0}$, we have

$$\begin{aligned} 0 =&\liminf_{k\rightarrow \infty} C_{\eta}\bigl( \Vert Ju_{n_{k+1}}-Ju_{n_{k}} \Vert _{V^{\ast}}+ \Vert u_{n_{k+1}}-u_{n_{k}} \Vert _{V}\bigr) \\ \geq& \liminf_{k\rightarrow \infty}h(u_{n_{k}})\langle w_{n_{k}},u_{n_{k}}-\eta\rangle+ \liminf_{k\rightarrow \infty} \bigl(F_{1}(u_{n{k}})-F_{1}(\eta)\bigr)+\liminf _{k\rightarrow \infty} \langle f,\eta -u_{n_{k}}\rangle \\ \geq& \bigl\langle h(\bar{w}),\bar{u}-\eta\bigr\rangle + F_{1}(\bar{u})-F_{1}(\eta)+\langle f,\eta-\bar{u}\rangle, \end{aligned}$$

where $\bar{w}\in A_{0}\bar{u}$. This means that $\bar{u}\in \mathit{SOL}(M,F_{1},h(w)-f)$.

*Step* 5. We prove that

3.24 $$\begin{aligned} \limsup_{k\rightarrow \infty} \bigl[\langle-J\bar{u},u_{n_{k+1}}-\bar{u} \rangle+ F_{1}(\bar{u})-F_{1}(u_{n_{k+1}}) \bigr]\leq0, \end{aligned}$$

where $\bar{u}=\Pi_{\mathit{SOL}(M, F_{1}, h(w)-f)}^{F_{1}} 0$.

Indeed take a subsequence $\{u_{n_{k+1}}\}$ of $\{u_{n}\}$ such that $u_{n_{k+1}}\rightharpoonup\bar{u}$.

Note that $\bar{u}=\Pi_{\mathit{SOL}(M, F_{1}, h(w)-f)}^{F_{1}} 0$. Then from $\bar{u}\in \mathit{SOL}(M, F_{1}, h(w)-f)$, the weak lower semicontinuity of $F_{1}$, and Lemma 2.1(ii), the desired inequality (3.24) follows immediately.

*Step* 6. We show that $\nonumber\lim_{n\rightarrow\infty} \Vert u_{n}-\bar{u} \Vert _{V}=0$.

Since $u_{n_{k+1}}\rightharpoonup\bar{u}$, it follows from the weak lower semicontinuity of $\Vert \cdot \Vert _{V}$ that

3.25 $$\begin{aligned} \liminf_{k\rightarrow\infty} \Vert u_{n_{k+1}} \Vert _{V}\geq \Vert \bar{u} \Vert _{V}. \end{aligned}$$

From the convexity of $D(F_{1})$, $F_{1}$ and from the weak lower semicontinuity of $F_{1}$, we obtain that $F_{1}$ is subdifferentiable in $\operatorname{int} (D(F_{1}))$. Thus, for all $u\in D(F_{1})$, there exists an element $u^{\ast}\in V^{\ast}$ such that

$$\begin{aligned} F_{1}(u)-F_{1}(\bar{u})\geq \bigl\langle u^{\ast},u-\bar{u}\bigr\rangle , \end{aligned}$$

and hence

3.26 $$\begin{aligned} F_{1}(u_{n_{k+1}})-F_{1}(\bar{u})\geq \langle Ju_{n_{k}},u_{n_{k+1}}-\bar{u}\rangle, \quad k\geq0. \end{aligned}$$

In view of $u_{n_{k+1}}=\Pi_{\mathit{SOL}(M, F_{1}, h(w)-f)}^{F_{1}} J^{-1}(Ju_{n_{k}}-\tau h(u_{n_{k}})(f-w_{n_{k}}))$, we have

$$\begin{aligned} G_{1}\bigl(u_{n_{k+1}}, Ju_{n_{k}}-\tau h(u_{n_{k}}) (f-w_{n_{k}})\bigr)\leq G_{1}\bigl(\bar{u}, Ju_{n_{k}}-\tau h(u_{n_{k}}) (f-w_{n_{k}})\bigr). \end{aligned}$$

By using (2.1) with $J\eta=Ju_{n_{k}}-\tau h(u_{n_{k}})(f-w_{n_{k}})$, we have

$$\begin{aligned}& \Vert u_{n_{k+1}} \Vert _{V}^{2}-2\bigl\langle Ju_{n_{k}}-\tau h(u_{n_{k}}) (f-w_{n_{k}}),u_{n_{k+1}} \bigr\rangle + \bigl\Vert Ju_{n_{k}}-\tau h(u_{n_{k}}) (f-w_{n_{k}}) \bigr\Vert _{V^{\ast}}^{2}+2F_{1}(u_{n_{k+1}}) \\& \quad \leq \Vert \bar{u} \Vert _{V}^{2}-2\bigl\langle Ju_{n_{k}}-\tau h(u_{n_{k}}) (f-w_{n_{k}}),\bar{u} \bigr\rangle \\& \qquad{}+ \bigl\Vert Ju_{n_{k}}-\tau h(u_{n_{k}}) (f-w_{n_{k}}) \bigr\Vert _{V^{\ast}}^{2}+2F_{1}( \bar{u}) \\& \quad = \Vert \bar{u} \Vert _{V}^{2}-2\bigl\langle Ju_{n_{k}}-\tau h(u_{n_{k}}) (f-w_{n_{k}}),u_{n_{k+1}} \bigr\rangle \\& \qquad {}+2\bigl\langle Ju_{n_{k}}-\tau h(u_{n_{k}}) (f-w_{n_{k}}),u_{n_{k+1}}-\bar{u}\bigr\rangle \\& \qquad {}+ \bigl\Vert Ju_{n_{k}}-\tau (u_{n_{k}}) (f-w_{n_{k}}) \bigr\Vert _{V^{\ast}}^{2}+2F_{1}( \bar{u}), \end{aligned}$$

which implies that

3.27 $$\begin{aligned} \Vert u_{n_{k+1}} \Vert _{V}^{2} \leq& \Vert \bar{u} \Vert _{V}^{2}+2 \bigl(\langle Ju_{n_{k}},u_{n_{k+1}}-\bar{u}\rangle +F_{1}( \bar{u})-F_{1}(u_{n_{k+1}})\bigr) \\ &{}+2\tau h(u_{n_{k}}) \langle w_{n_{k}},u_{n_{k+1}}-\bar{u} \rangle+2\tau h(u_{n_{k}})\langle f,\bar{u}-u_{n_{k+1}}\rangle. \end{aligned}$$

Taking the $\limsup_{k\rightarrow\infty}$ on the both sides of (3.27) and using $u_{n_{k+1}}\rightharpoonup\bar{u}$, (3.22)-(3.24), and (3.26) yields

$$\begin{aligned} \limsup_{k\rightarrow\infty} \Vert u_{n_{k+1}} \Vert _{V}^{2}\leq \Vert \bar{u} \Vert _{V}^{2}, \end{aligned}$$

which implies that

3.28 $$\begin{aligned} \limsup_{k\rightarrow\infty} \Vert u_{n_{k+1}} \Vert _{V}\leq \Vert \bar{u} \Vert _{V}. \end{aligned}$$

Combining (3.25) and (3.28), we have

3.29 $$\begin{aligned} \Vert \bar{u} \Vert _{V}\leq\liminf_{k\rightarrow \infty} \Vert u_{n_{k+1}} \Vert _{V}\leq\limsup_{k\rightarrow \infty} \Vert u_{n_{k+1}} \Vert _{V}\leq \Vert \bar{u} \Vert _{V}. \end{aligned}$$

This shows that

3.30 $$\begin{aligned} \lim_{k\rightarrow\infty} \Vert u_{n_{k+1}} \Vert _{V} = \Vert \bar{u} \Vert _{V}. \end{aligned}$$

Since *V* is a uniformly convex Banach space, then it has the Kadec-Klee property, and so from $u_{n_{k+1}}\rightharpoonup\bar{u}$ and (3.30) we obtain

3.31 $$\begin{aligned} \lim_{k\rightarrow\infty} u_{n_{k+1}} = \bar{u}. \end{aligned}$$

Let us now show that the whole sequence converges strongly to *ū*.

Since $\{G_{2}(u_{n+1},0)\}$ is bounded and nondecreasing (indeed, by Lemma 2.2(i), we have $G_{2}(u_{n+1},u_{n})+G_{2}(u_{n+1},0)\leq G_{2}(u_{n},0)$ and $G_{2}(u_{n+1},u_{n})\geq ( \Vert u_{n+1} \Vert _{V}- \Vert u_{n} \Vert _{V})^{2}\geq 0$), it follows that $\{G_{2}(u_{n+1},0)\}$ is convergent.

This together with (3.31) implies that

3.32 $$\begin{aligned} \lim_{n\rightarrow\infty}G_{2}(u_{n+1},0)=G_{2}( \bar{u},0). \end{aligned}$$

Now, following to [31], we suppose that there exists some subsequence $\{u_{n_{j+1}}\}$ of $\{u_{n}\}$ such that $\lim_{j\rightarrow \infty} u_{n_{j+1}} = \hat{u}$, then by Lemma 2.2(i) we obtain

$$\begin{aligned} 0\leq G_{2}(\bar{u},\hat{u}) =&\lim_{k,j \rightarrow \infty}G_{2}(u_{n_{k+1}},u_{n_{j+1}})= \lim_{k,j \rightarrow \infty}G_{2}\bigl(u_{n_{k+1}}, \Pi_{\mathit{SOL}(M, F_{1}, h(w)-f)}^{F_{1}}0\bigr) \\ \leq&\lim_{k,j \rightarrow \infty} \bigl[G_{2}(u_{n_{k+1}},0)-G_{2} \bigl(\Pi_{\mathit{SOL}(M, F_{1}, h(w)-f)}^{F_{1}}0,0\bigr) \bigr] \\ =&\lim_{k,j \rightarrow \infty} \bigl[G_{2}(u_{n_{k+1}},0)-G_{2}(u_{n_{j+1}}, 0) \bigr] \\ =&G_{2}(\bar{u},0)-G_{2}(\bar{u},0)=0, \end{aligned}$$

which means that $G_{2}(\bar{u}, \hat{u})=0$ and hence, by Lemma 2.2(ii), it results that $\hat{u}=\bar{u}$.

Consequently, $\lim_{n\rightarrow\infty} u_{n}=\bar{u}$. This completes the proof of Theorem 3.1.

#### Theorem 3.2

*Let* $V=H$ *be a real Hilbert space*, *and let* *M* *be a nonempty closed convex subset of* *H*. *Let* $A_{0}:H \rightarrow C(H)$ *be a multivalued mapping*. *Suppose that* $A_{0}$ *is a pseudomonotone*, *coercive*, *potential*, *and inverse strongly monotone mapping*. *Let* $\{h(u_{n})\}$, *M*, *F̃*, $\tilde{{S}_{0}}$, $\tilde{\mu}_{0}$ *and* $\tilde{F}_{0}$, $F_{1}$, $\tilde{R}_{0}$, $\tilde{R}_{1}$ *be the same as in Theorem* 3.1.

*Then*, *for arbitrary* $u_{0}=u\in M$, *the sequence* $\{u_{n}\}$ *defined by*

3.33 $$\begin{aligned} (u_{n+1}-u_{n},\eta-u_{n+1})+\tau \bigl(F_{1}(\eta)-F_{1}(u_{n+1})\bigr)\geq\tau \bigl(f-h(u_{n})w_{n},\eta-u_{n+1}\bigr) \quad \forall \eta\in M, \end{aligned}$$

*with* $0<\tau<\tau_{0}=\frac{2\gamma}{h}$, $h>0$, *converges strongly to* $\tilde{u}=\Pi_{\mathit{SOL}(M,F_{1},h(w)-f)}^{F_{1}}{0}$.

#### Proof

Since any inverse strongly monotone mapping is $\frac{1}{\gamma}$-Lipschitzian mapping, i.e., bounded Lipschitz continuous with $\mu(\xi)=\frac{1}{\gamma_{0}}$ and $\Phi(\xi )=\xi$, then by simple modifications of the proof of Theorem 3.1, we can easily show that there exists a subsequence $\{u_{n_{k+1}}\}$ of $\{u_{n}\}$ such that $u_{n_{k+1}} \rightharpoonup\bar{u}\in \mathit{SOL}(M,F_{1}, h(w)-f)$ and $\lim_{k\rightarrow\infty} \Vert u_{n_{k+1}} \Vert _{H} = \Vert \bar{u} \Vert _{H}$.

Since every Hilbert space is uniformly convex, by virtue of the Kadec-Klee property of *H*, we have $\lim_{k\rightarrow\infty} u_{n_{k+1}}=\bar{u}\in \mathit{SOL}(M,F_{1}, h(w)-f)$.

Now, we prove that $u_{n}\rightharpoonup\bar{u}$ and $\lim_{n\rightarrow\infty} \Vert u_{n} \Vert _{H} = \Vert \bar{u} \Vert _{H}$.

From $\bar{u}\in \mathit{SOL}(M,F_{1}, h(w)-f)$, we have

3.34 $$\begin{aligned} \tau\bigl(F_{1}(\eta)-F_{1}(\bar{u})\bigr)\geq\tau \bigl(f-h(\bar{w}),\eta-\bar{u}\bigr), \quad \forall\eta\in M. \end{aligned}$$

Setting $\eta=u_{n+1}$ in (3.34) and $\eta=\bar{u}$ in (3.33), we have

3.35 $$\begin{aligned} \tau\bigl(F_{1}(u_{n+1})-F_{1}(\bar{u})\bigr)\geq \tau \bigl(f-h(\bar{w}),u_{n+1}-\bar{u}\bigr) \end{aligned}$$

and

3.36 $$\begin{aligned} \tau\bigl(F_{1}(\bar{u})-F_{1}(u_{n+1})\bigr)\geq (u_{n+1}-u_{n},u_{n+1}-\bar{u})+\tau \bigl(f-h(u_{n})w_{n},\bar{u}-u_{n+1}\bigr). \end{aligned}$$

Adding (3.35) and (3.36), we have

$$\begin{aligned} (u_{n+1}-\bar{u},u_{n+1}-\bar{u}) \leq&(u_{n}-\bar {u},u_{n+1}-\bar{u}) -\tau \bigl(h(u_{n})w_{n}-h(\bar{w}),u_{n+1}-\bar{u} \bigr) \\ =&\bigl(u_{n}-\bar{u}-\tau\bigl(h(u_{n})w_{n}-h( \bar {w})\bigr),u_{n+1}-\bar{u}\bigr), \end{aligned}$$

which implies that

$$\begin{aligned} \Vert u_{n+1}-\bar{u} \Vert _{H}\leq \bigl\Vert u_{n}-\bar{u}-\tau \bigl(h(u_{n})w_{n}-h( \bar{w})\bigr) \bigr\Vert _{H}. \end{aligned}$$

Then, by the inverse strong monotonicity of $A_{0}$, we obtain for all sufficiently large *n*

$$\begin{aligned} \Vert u_{n+1}-\bar{u} \Vert _{H}^{2} \leq& \Vert u_{n}-\bar{u} \Vert _{H}^{2}-2\tau \bigl(h(u_{n})w_{n}-h(\bar{w}),u_{n}-\bar{u} \bigr)+\tau^{2} \bigl\Vert h(u_{n})w_{n}-h( \bar{w}) \bigr\Vert _{H}^{2} \\ =& \Vert u_{n}-\bar{u} \Vert _{H}^{2}-2 \tau h (w_{n}-\bar{w},u_{n}-\bar{u})+\tau^{2}h^{2} \Vert w_{n}-\bar{w} \Vert _{H}^{2} \\ \leq& \Vert u_{n}-\bar{u} \Vert _{H}^{2}- \tau h\biggl(2-\frac{\tau h}{\gamma}\biggr) \Vert w_{n}-\bar{w} \Vert _{H}^{2}. \end{aligned}$$

Since $2-\frac{\tau h}{\gamma}>0$, it follows that $\Vert u_{n+1}-\bar{u} \Vert _{H}\leq \Vert u_{n}-\bar{u} \Vert _{H}$ and so $\lim_{n\rightarrow \infty} \Vert u_{n}-\bar{u} \Vert _{H}=\sigma_{\bar{u}}$.

By following the same arguments as in [1] and [32], we can readily claim that all weak limit points of the sequence $\{u_{n}\}$ coincide, and hence $u_{n}\rightharpoonup\bar{u}$ as $n\rightarrow\infty$.

By the weak lower semicontinuity of $\Vert \cdot \Vert _{H}$, this implies that

3.37 $$\begin{aligned} \liminf_{n\rightarrow\infty} \Vert u_{n} \Vert _{H} > \Vert \bar{u} \Vert _{H}. \end{aligned}$$

Analogically to the proof of step 6 with obvious modifications, we have

3.38 $$\begin{aligned} \limsup_{n\rightarrow\infty} \Vert u_{n} \Vert _{H} \leq \Vert \bar{u} \Vert _{H}. \end{aligned}$$

This, together with (3.37), implies that $\lim_{n\rightarrow \infty} \Vert u_{n} \Vert _{H} = \Vert \bar{u} \Vert _{H}$.

Applying again the virtue of the Kadec-Klee property of *H*, we obtain $\lim_{n\rightarrow\infty} u_{n}= \bar{u}$. This completes the proof of Theorem 3.2. □

#### Remark 3.1

Theorems 3.1 and 3.2 extend and improve the corresponding Theorems 1.1 and 1.2.

#### Example 3.1

Axisymmetric shell problem

A quintessential example of a single-valued mapping satisfying all the assumptions contemplated in Theorems 3.1 and 3.2 which appears in determining the axisymmetric equilibrium position of a soft netlike rotation shell is as follows:

The shell surface (in a strainless state) is assumed to be a cylinder of length *l* and radius 1. Let *s* be a Lagrangian coordinate in the longitudinal direction such that $0< s< l$.

Let $V=[\overset{\circ}{W}{}_{p}^{(1)}(0,l)]^{2}$ and $V^{\ast}=[\overset{\circ}{W}{}_{q}^{(-1)}(0,l)]^{2}$, $q=\frac{p}{p-1}$, $p>1$. Set $u(s)=(u_{1}(s),u_{2}(s))$, $\eta(s)=(\eta_{1}(s),\eta_{2}(s))$, $M=\{u\in V:u_{2}(s)+1\geq0\ \forall s\in(0,l)\}$, and $\lambda_{1}=[(1+\frac{du_{1}}{ds})^{2}+(\frac {du_{2}}{ds})^{2}]^{\frac{1}{2}}$, $\lambda_{2}=1+u_{2}$.

Consider the surface force is characterized by a known constant function $\mathbb{P}$. Let $T_{i}(\lambda_{i})$, $i=1,2$, be two functions (tightening force) satisfying conditions (3)-(5) in Badriev and Banderov [33].

Consider the mappings $A,B,C,D: V\rightarrow V^{\ast}$ defined by

$$\begin{aligned}& \langle Au, \eta\rangle= \int_{0}^{l}\frac{T_{1}(\lambda_{1})}{\lambda_{1}} \biggl(\biggl(1+ \frac{du_{1}}{ds},\frac{du_{2}}{ds}\biggr), \frac{d\eta}{ds}\biggr) \,ds; \\& \langle Bu, \eta\rangle= \int_{0}^{l}\biggl(\frac{1}{2} u_{2}^{2} \frac{d\eta_{1}}{ds}+\biggl(1+\frac{du_{1}}{ds} \biggr) u_{2} \eta_{2}\biggr) \,ds; \\& \langle Cu, \eta\rangle= \int_{0}^{l}\biggl(\biggl(1+\frac{du_{1}}{ds} \biggr) \eta_{2}+\frac {du_{2}}{ds} \frac{d\eta_{1}}{ds}\biggr) \,ds; \\& \langle Du, \eta\rangle= \int_{0}^{l}{T_{2}(\lambda_{2})} \eta_{2} \,ds. \end{aligned}$$

If $A_{0}=(A+D)+\mathbb{P}(B+C)$, then by Theorems 2 and 3 in [33] it follows that the mapping $A_{0}$ satisfies all the assumptions postulated in Theorems 3.1 and 3.2.

## Conclusion

A generalized multivalued pseudomonotone mixed variational inequality is considered, and a modified two-layer iteration via a boundary point approach to find the minimum norm solution of such inequalities is introduced, and its strong convergence is proved in the framework of uniformly convex spaces. The results develop the corresponding recent results.
